# Ets1-Related Protein Is a Key Regulator of Vasculogenesis in Zebrafish

**DOI:** 10.1371/journal.pbio.0040010

**Published:** 2005-12-20

**Authors:** Saulius Sumanas, Shuo Lin

**Affiliations:** **1**Department of Molecular, Cell, and Developmental Biology, University of California, Los Angeles, United States of America; Wellcome Trust Sanger InstituteUnited Kingdom

## Abstract

During embryonic development, multiple signaling pathways control specification, migration, and differentiation of the vascular endothelial cell precursors, angioblasts. No single gene responsible for the commitment of mesenchymal cells to the angioblast cell fate has been identified as yet. Here we report characterization and functional studies of Etsrp, a novel zebrafish ETS domain protein. *etsrp* embryonic expression is only restricted to vascular endothelial cells and their earliest precursors. Morpholino knockdown of Etsrp protein function resulted in the complete absence of circulation in zebrafish embryos. Angioblasts in *etsrp*–morpholino-injected embryos (morphants) failed to undergo migration and differentiation and did not coalesce into functional blood vessels. Expression of all vascular endothelial molecular markers tested was severely reduced in *etsrp* morphants, whereas hematopoietic markers were not affected. Overexpression of *etsrp* RNA caused multiple cell types to express vascular endothelial markers. *etsrp* RNA restored expression of vascular markers in *cloche* mutants, defective in hematopoietic and endothelial cell formation, arguing that *etsrp* functions downstream of *cloche* in angioblast formation. *etsrp* gene function was also required for endothelial marker induction by the *vascular endothelial growth factor (vegf)* and *stem cell leukemia (scl/tal1).* These results demonstrate that Etsrp is necessary and sufficient for the initiation of vasculogenesis.

## Abstract 2

The authors report on a novel protein, Ets1 related protein (Etsrp), which is both necessary and sufficient for the development of the endothelial cells that line blood vessels (angioblasts).

## Introduction

Vasculogenesis, or formation of vascular endothelial cells de novo, begins very early after the initiation of gastrulation in a vertebrate embryo. In a mammalian embryo, vasculogenesis starts with the formation of the blood islands in the yolk sac and angioblast precursors in the head mesenchyme [1]. In the zebrafish, the first angioblasts arise from the lateral plate mesoderm, migrate to the trunk midline between ten- and 15-somite stages in response to *hedgehog* signaling, and coalesce to form the primary axial vessels of the trunk, the dorsal aorta, and the cardinal vein [2–4]. During subsequent angiogenesis, the axial vessels sprout to form secondary vessels in the trunk region of a zebrafish embryo [5]. In the zebrafish, as well as the mammalian yolk sac, there is a close association between the primitive hematopoietic cells and the developing endothelium, suggestive of a common precursor, the hemangioblast [6]. Zebrafish *cloche* mutants lack nearly all blood and endothelial cells suggesting that the hemangioblast lineage has been affected [7,8]. A basic helix-loop-helix transcription factor Scl/tal1 is expressed in hematopoietic and endothelial cells in both mouse and zebrafish suggesting a possible role in specification of the hemangioblast [9–12]. Recent knockdown analysis has demonstrated its essential function in blood and dorsal aorta formation in zebrafish embryos [13,14].

Members of the Ets family of transcription factors play multiple roles during vasculogenesis, angiogenesis, and hematopoiesis [15]. They share a conserved DNA-binding domain of 85 amino acids which folds into a winged helix-turn-helix motif. Ets1, the founding member of the family, is expressed in the embryonic endothelial cells as well as putative hemangioblasts, lymphoid precursors, and myeloid hematopoietic cells [16–19]. The expression of Ets1 transcripts is associated in vivo with the activation of endothelial cells and the induction of angiogenesis. In vitro, Ets1 is expressed by proliferating and migrating endothelial cells but not after these cells have reached confluence [20,21]. In endothelial cells in vitro, Ets1 has been shown to regulate expression of the two vascular endothelial growth factor (Vegf) receptors Flt1 and Flk1 [22,23], the endothelial cell-specific adhesion protein VE-cadherin [24], the vascular-specific tyrosine kinases Tie1 and Tie2 [25], and numerous other target genes [15]. Ets1 antisense oligonucleotides inhibited angiogenesis in vitro [26,27]. However, no defects in vascular development have been found in the mouse embryos, nearly completely deficient in Ets1 function [28,29]. Therefore, the function of Ets1 in vascular development in vivo, if any, remains unclear.

A different Ets family member Fli1 has been implicated in mouse megakaryopoiesis, hemostasis, and maintenance of vascular integrity [30,31]. Zebrafish *fli1* homolog is expressed during early somitogenesis in the putative hemangioblast cells, while later being restricted to vascular endothelial cells [32].

Recently, an endothelial-cell-specific Ets1-related zebrafish protein Etsrp has been identified [33]. In the current study, we demonstrate a critical role of Etsrp during vasculogenesis. In the absence of Etsrp, angioblast precursors fail to migrate, differentiate, and coalesce into functional vessels. Furthermore, ectopic expression of *etsrp* is sufficient to induce endothelial markers in a variety of different cell types throughout the embryo. These results suggest that Etsrp functions as a critical regulatory gene, directing *etsrp-*expressing cells to adopt vascular endothelial cell fate during embryonic development.

## Results

### Etsrp Encodes a Novel Ets1-Related Protein

**Figure 1:**
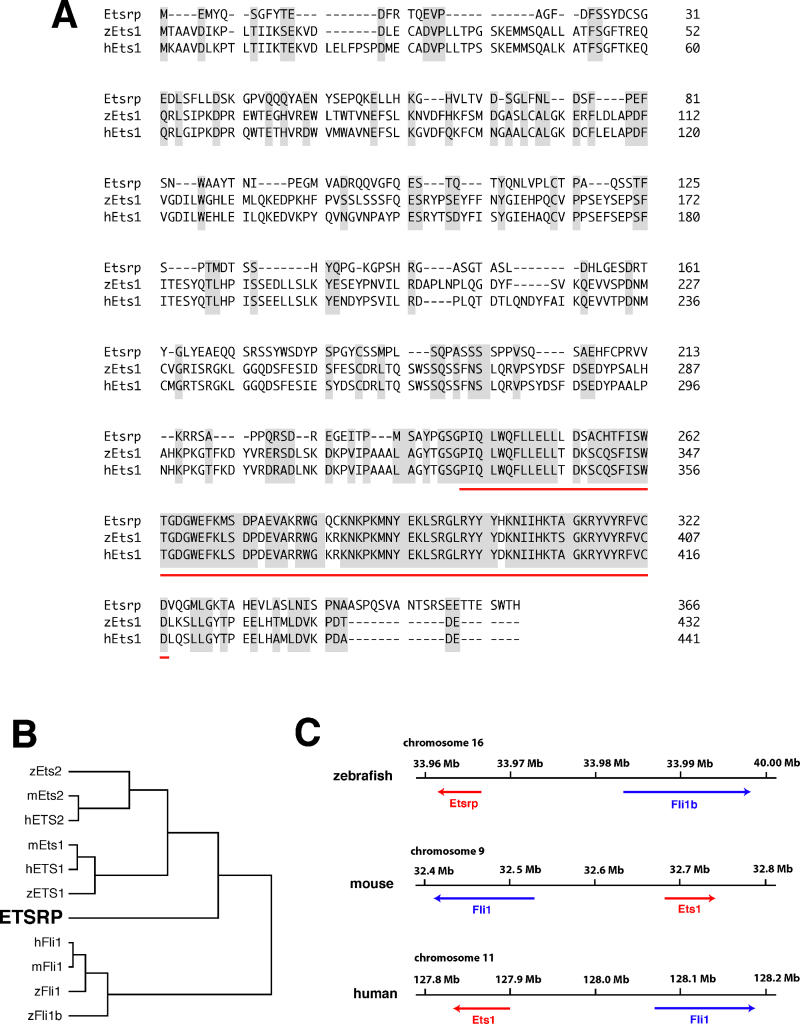
Sequence Analysis of the Zebrafish Ets1-Related Protein Etsrp (A) Shows alignment of Etsrp and its closest human and zebrafish homologs Ets1 proteins. Etsrp and hEts1 share 88% homology within the ETS DNA-binding domain (underlined in red), and a very limited homology within the rest of the sequence. Identical and similar amino acids are shaded in grey. (B) The homology tree of Etsrp and its closest human, mouse, and zebrafish homologs. Length of horizontal branches is directly proportional to the evolutionary distance between the proteins. Zebrafish Ets1 and Ets2 protein sequences have been predicted using the available EST sequences TC282499 and TC270146 (http://www.tigr.org). GeneWorks 2.5 has been used to build the alignment and the homology tree. (C) Chromosomal location of the zebrafish *etsrp,* mouse, and human *ets1* genes. In all cases, they are positioned next to a *fli1* homolog.

A novel Ets1-related protein Etsrp has been identified in a recent microarray study of the zebrafish *cloche* mutants [33]. We performed detailed analysis of the protein sequence and its developmental expression pattern. Etsrp-predicted protein sequence contains 366 amino acids and displays 29% identity (37% similarity) to the human Ets1 protein and 26% identity (34% similarity) to the human Ets2 (Figure 1A) (unpublished data). Although Etsrp and hEts1 proteins are 88% similar within the ETS domain region (Figure 1A), there is little similarity between the two proteins within the rest of the sequence, therefore Etsrp may not be a functional ortholog of the Ets1 protein. Supporting this idea, a recently published protein sequence of the zebrafish, Ets1 homolog, was found to be highly similar to the Ets1 subfamily throughout most of the protein sequence (Figure 1A) [34]. No other homology domains were found outside the ETS domain of Etsrp. We were unable to find an apparent Etsrp ortholog in other species. As evident from the homology tree, Etsrp is the most closely related to Ets1 and Ets2 subfamilies (Figure 1B). We performed synteny analysis of human, mouse, and zebrafish *ets1* or *etsrp* chromosomal regions. Human and mouse *ets1* genes are positioned next to *fli1* genes, which encode related ETS domain proteins (Figure 1C). *ets1* and *fli1* genes are transcribed in opposite directions and are likely to have originated from the same ancestral precursor via gene duplication. Zebrafish *etsrp* is also positioned in the opposite direction and next to a *fli1*-related gene *fli1b* [34]. This analysis suggests that *etsrp* is evolutionarily related to the mammalian *ets1* genes. As the zebrafish genome has undergone an additional duplication [35], *etsrp* and *ets1* may have diverged from each other and acquired separate functions. Alternatively, it is possible that *ets1* duplicated prior to the divergence of teleosts and tetrapods, and *etsrp* was subsequently lost in the tetrapod lineage.

### Etsrp Expression Pattern Analysis

**Figure 2:**
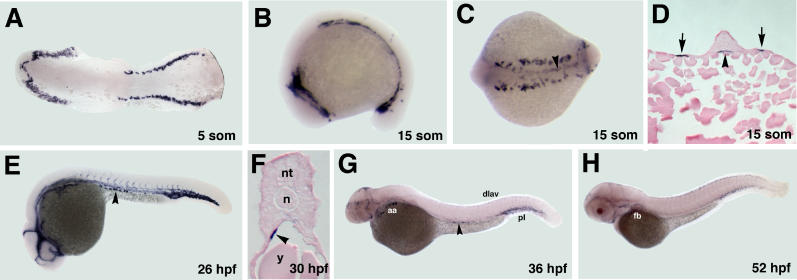
Expression Pattern of *etsrp* as Analyzed by In Situ Hybridization Anterior is to the left except as noted. (A) five-somite stage. *etsrp* is expressed within lateral mesoderm in two distinct expression domains, in the anterior and posterior parts of an embryo. (B) 15-somite stage, lateral view. (C) dorsal view. (D) transverse section. *etsrp* is expressed in two bilateral stripes of presumptive angioblasts within the lateral mesoderm in the anterior and the trunk and posterior parts of an embryo (arrows, D). In addition, a stripe of *etsrp*-expressing cells is apparent at the midline and extends through the middle and posterior parts of an embryo (arrowhead, C and D). (E) 26 hpf stage. *etsrp* is expressed in vascular endothelial cells of the axial, head, and intersomitic vessels. Note a group of *etsrp*-expressing cells bilaterally located in the intermediate mesoderm (arrowhead). (F) Transverse section through the trunk region of a 30 hpf embryo. Arrowhead shows one of the *etsrp*-expressing cells located within the lateral/intermediate mesoderm. Expression of *etsrp* in the axial vessels is weak at this stage and not apparent in this section. nt, neural tube; n, notochord; y, yolk. (G) 36 hpf stage. *etsrp* is expressed in a subset of head vessels, the aortic arches (aa), the cardinal vein plexus (pl) region, posterior intersomitic vessels, and the dorsal longitudinal anastomotic vessel (dlav). Note a group of *etsrp*-expressing cells located in the endodermal region (arrowhead). (H) 52 hpf stage. *etsrp* expression is observed in a subset of head vessels, common cardinal vein, pectoral fin bud blood vessels (fb), cardinal vein plexus region, and weakly in posterior intersegmental vessels and the dorsal vessel.

We analyzed the expression pattern of *etsrp* RNA in early embryos using in situ hybridization. No expression was observed prior to the one-somite stage (unpublished data). From the two-somite stage onward, *etsrp* RNA was localized to two stripes of cells within the lateral mesoderm (five-somite embryo, Figure 2A). Two distinct expression domains, in the anterior and posterior parts of an embryo, were apparent. The anterior stripes merged at the prechordal plate. The *etsrp*-expressing cells are likely endothelial cell precursors based on the expression of the gene at later stages. At the 15-somite stage, expression in two bilateral stripes of cells within the lateral mesoderm in the anterior and middle/posterior parts of an embryo, was apparent (Figure 2B–2D). In addition, a stripe of *etsrp*-expressing cells was noted at the midline and extended through the trunk and posterior parts of an embryo. The middle stripe likely represents the endothelial precursor cells that have already migrated to the future intermediate cell mass (ICM) region. By 26 hours-post-fertilization (hpf), *etsrp* was expressed in vascular endothelial cells in the embryo marking main axial, head, and intersegmental vessels (Figure 2E). In addition, a group of *etsrp*-expressing cells was located in the intermediate mesoderm region (Figure 2E and 2F). These cells which by 36 hpf were located close to the pronephros (Figure 2G), and appeared to migrate posteriorly, may represent endothelial cell precursors of pronephric vessels and/or gut vessels. By 36 hpf *etsrp* expression had mostly disappeared from the axial vessels and was prominent in a subset of head vessels, aortic arches, the cardinal vein plexus region, posterior intersomitic vessels, and the dorsal longitudinal anastomotic vessel (Figure 2G). At 52 hpf, *etsrp* expression was observed in a subset of head vessels, the common cardinal vein, blood vessels of the pectoral fin, the cardinal vein plexus region, and weakly in posterior intersegmental vessels and the dorsal vessel (Figure 2H).

### Morpholino Knockdown of Etsrp Function Results in Loss of Circulation

**Figure 3:**
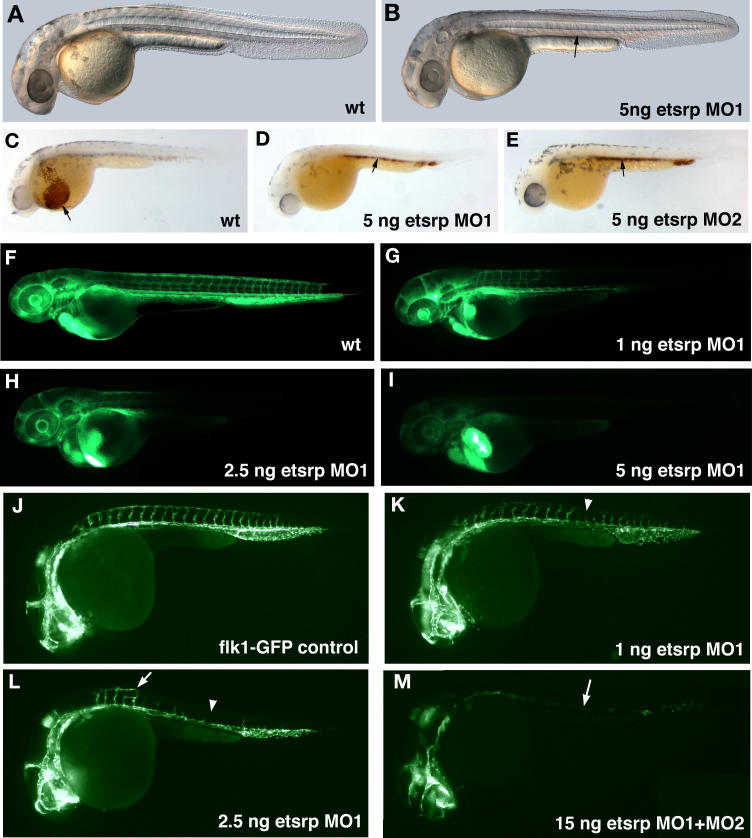
MO Knockdown of Etsrp Protein Function Disrupts Blood Vessel Formation in the Zebrafish Embryos (A,B) Morphological analysis of live *etsrp* morphants at 34 hpf. (A) Uninjected control embryo. (B) 5 ng of *etsrp* MO1-injected embryo. Notice that red blood cells are scattered throughout the circulatory system in the control uninjected embryo while they accumulate at their formation site within the intermediate cell mass (arrow, B) in the *etsrp* morphant. (C–E) o-dianisidine staining of heme in the red blood cells of uninjected control (C), 5 ng of *etsrp* MO1-injected (D) and 5 ng of *etsrp* MO2-injected (E) embryos at 34 hpf. While many circulating blood cells are apparent within the common cardinal vein before entering the heart in the control embryo (arrow, C), they stay at their formation site within the ICM region in *etsrp* morphants (arrows). (F–I) Microangiography analysis of the circulatory system by injecting fluorescein-labeled dextran into the sinus venosus of *etsrp* morphants at 55 hpf. (F) Control uninjected, (G) 1 ng of *etsrp* MO1-injected (H) 2.5 ng of *etsrp* MO1-injected (I) 5 ng of *etsrp* MO1-injected embryos. Note that the embryo in (G) has lost circulation in the posterior vessels, the embryo in (H) has lost circulation in most vessels, and the embryo in (I) has no circulation at all. (J–M) Analysis of blood vessels in live *flk1*-GFP transgenic embryos at 26 hpf. (J) Control uninjected, (K) 1 ng of *etsrp* MO1-injected, (L) 2.5 ng of *etsrp* MO1-injected, (M) 15 ng of *etsrp* MO1+MO2 (1:1) mix-injected embryos. Note the gaps in formation of intersegmental vessels in (K) (arrowhead), the missing (arrowhead) and abnormally branched, (arrow) intersegmental vessels in (L), and the nearly completely eliminated *flk1* expression from axial vessels (arrow) in (M).

We used antisense morpholino (MO) oligonucleotides [36,37] to knockdown the function of Etsrp. Etsrp–MO-injected embryos (morphants) showed no apparent circulation. While red blood cells were actively circulating in wild-type (wt) embryos at 34 hpf, in etsrp morphants they stayed at their formation site, the ICM region (Figure 3A and 3B). This observation was confirmed by the o-dianisidine staining of heme in the red blood cells (Figure 3C–3E). We performed microangiography analysis by injecting fluorescein-labeled high molecular weight dextran into the sinus venosus of the 2-day-old embryos. Lower doses of etsrp MOs caused partial loss of circulation in the intersegmental and axial vessels, particularly, in the posterior part of the embryo (Figure 3F and 3G). Head circulation was not affected at this dose (Figure 3G). Higher doses of etsrp MOs resulted in the complete loss of functional blood vessels (Figure 3H and 3I, Table 1). Other than the described defects, etsrp morphants appeared morphologically normal (Figure 3B). At 2–3 days-post-fertilization (dpf), etsrp morphants developed pericardial edema and eventually became necrotic and died (unpublished data). Injection of two different etsrp-specific translation-blocking MOs resulted in similar circulatory defects, demonstrating specificity of the observed phenotype (Table 1). To confirm that etsrp MOs were specifically inhibiting synthesis of Etsrp protein, we generated etsrp–green fluorescent protein (GFP) modified bacterial artificial chromosome (BAC). BAC construct of approximately 250 kilobases containing *etsrp* gene was modified using recA-mediated homologous recombination to replace *etsrp* coding sequence with GFP [38]. Microinjection of etsrp-GFP BAC resulted in the mosaic GFP expression (Figure S1A). Etsrp MO2, directed against the 5′ UTR of *etsrp*, completely inhibited this transient GFP expression (Figure S1B). Control injection of a 5-base mismatch MO had no effect on GFP expression (Figure S1C). These results confirm that etsrp MOs specifically inhibit *etsrp* gene function. Microinjection of a low dose of etsrp MO into flk1-GFP transgenic embryos [39] resulted in partial loss of *flk1* expression in intersegmental vessels (Figure 3J–3L). High doses of etsrp MOs resulted in nearly complete loss of *flk1* expression in axial vessels and strong downregulation of its expression in head vessels (Figure 3M).

**Table 1:**
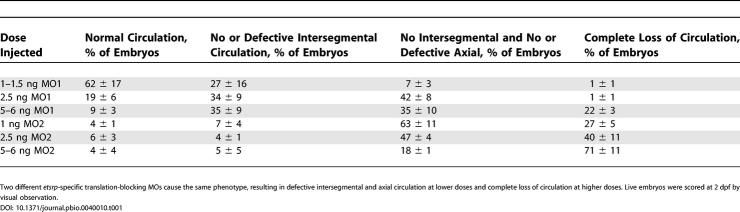
Dose Dependence of Etsrp MO Phenotype

### Vascular Endothelial Markers Are Lost in Etsrp Morphants

**Figure 4:**
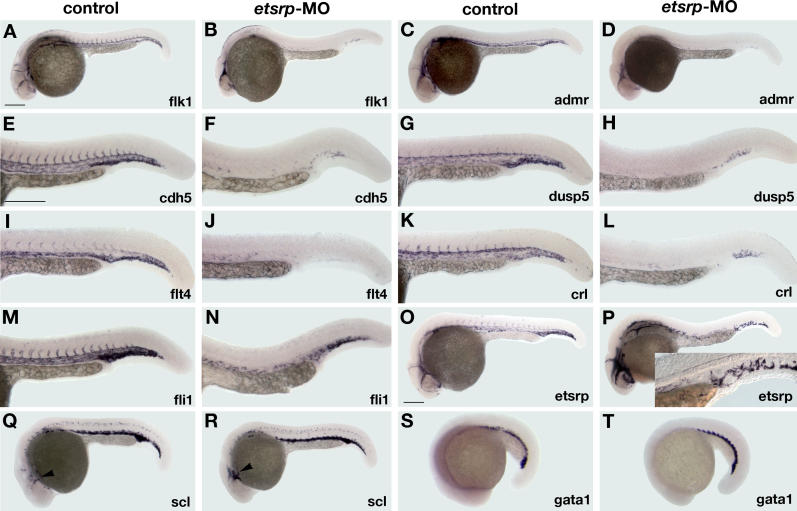
Molecular Analysis of Vascular Endothelial and Hematopoietic Markers in *etsrp* Morphants (A, C, E, G, I, K, M, O, Q, S) Uninjected control embryo; (D, H, J, L, P, R, T) 8 ng *etsrp* MO2-injected embryo; (B, F, N) 12 ng *etsrp* MO1+MO2 (1:1) mix-injected embryos for the maximum knockdown. All embryos are at 24 hpf, except as noted. Scale bar, 0.2 mm. (A,B) *flk1* expression; (C,D) *admr* expression; (E,F) *cdh5* expression; (G,H) *dusp5* expression; (I,J) *flt4* expression; (K,L) *crl* expression. Note that the vascular expression of the markers in (A–L) is almost absent in *etsrp* morphants. (M,N) *fli1* expression; (O,P) *etsrp* expression. Note the more intense *etsrp* expression and reduced *fli1* expression in angioblasts which remain dispersed and fail to coalesce into blood vessels in *etsrp* morphants. Inset, (P) DIC image of scattered angioblasts in *etsrp* morphants. (Q,R) *scl* expression, 22 hpf. Note that *scl* expression appears unaffected at this stage except for more intense staining in a subset of head vessels in *etsrp* morphants (arrowhead). (S,T) *gata1* expression, 22 somites. Note that no significant difference in hematopoietic *gata1* expression is observed between the control embryos and *etsrp* morphants.

We performed analysis of different molecular markers in *etsrp* morphants using high doses of *etsrp* MOs. *Kinase insert domain receptor (kdr, flk1)* [40], *cadherin 5 (cdh5, VE-cadherin)* [41], *adrenomedullin receptor (admr)* [42], *dual specificity phosphatase 5 (dusp5)* [42], and *C1qR-like (crl)* [42] genes are expressed in all vascular endothelial cells while *fms-related tyrosine kinase 4 (flt4)* [40] is restricted to the venous vessels in early zebrafish embryos. Expression of all of these markers in axial and intersegmental vessels was nearly completely abolished in *etsrp* morphants at 24 hpf (Figure 4A–4L). Head vessel expression, however, was reduced but still apparent even at high MO doses (Figure 4A–4D) (unpublished data). We were unable to use even higher MO doses to attempt to completely eliminate Etsrp function due to mild toxic effects observed at doses above 15 ng (unpublished data).

Expression of *friend leukemia integration 1 (fli1)* [40] was downregulated but still apparent in *etsrp* morphants (Figure 4M and 4N). Interestingly, expression of *etsrp* itself was upregulated in *etsrp* morphants, suggesting the presence of a negative autoregulatory loop (Figure 4O and 4P). However, the number of *etsrp*-expressing angioblasts was reduced in *etsrp* morphants. These angioblasts remained scattered within the lateral mesoderm failing to coalesce into functional blood vessels (Figure 4P). Formation of hematopoietic precursors appeared unaffected as evident from *scl* and *gata1* expression (Figure 4Q–4T).

**Figure 5:**
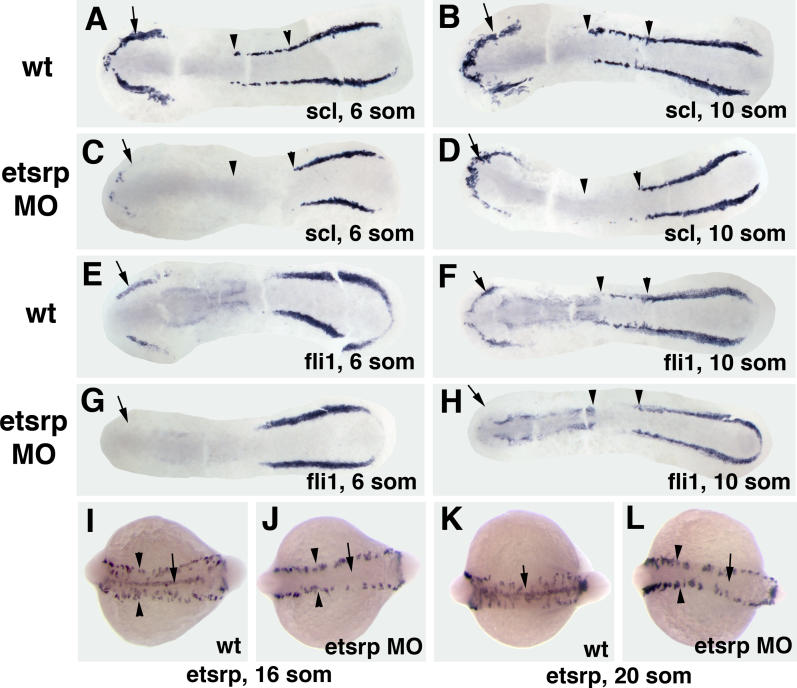
Molecular Analysis of Early Vasculogenesis in *etsrp* Morphants (A, B, E, F, I, K) Uninjected control embryo; (C, D, G, H, J, L) 8–10 ng *etsrp* MO2-injected embryo. Anterior is to the left in all panels. (A–H) Embryos were flat mounted with their yolk removed. (A–D) *scl* expression; six-somite (A,C) and ten-somite (B,D) stages. Note that the anterior domain of *scl* expression (arrows) is reduced and the trunk domain (arrowheads) is missing in *etsrp* morphants. (E–H) *fli1* expression; six-somite (E,G) and ten-somite (F,H) stages. Note that the anterior domain of *fli1* expression (arrows) is missing in the *etsrp* morphants, while the posterior domain is not affected. Also note that the trunk domain of *fli1* expression (arrowheads, F,H) is missing at the ten-somite stage in *etsrp* morphants. (I–L) Etsrp knockdown blocks angioblast migration towards the midline as assayed by *etsrp* expression at the 16-somite (I,J) and 20-somite (K,L) stages. (I,K) Uninjected control embryo; (J,L) 7.5 ng *etsrp* MO2-injected embryo. Dorsal view, anterior is to the left. Note that the midline stripe of angioblasts (arrows) is missing in *etsrp* morphants. Also notice more intense *etsrp* expression in pre-migratory angioblasts (arrowheads) in *etsrp* morphants as compared to control embryos.

We analyzed formation of hematopoietic and vascular progenitors during early somitogenesis stages. A transcription factor *scl* is expressed in both hematopoietic and vascular endothelial cell precursors [12]. Expression of *scl* in *etsrp* morphants was reduced in the anterior domain and absent from the trunk domain while its posterior expression was not affected at the six- and ten-somite stages (Figure 5A–5D). *fli1* is expressed in the putative hemangioblast cells while later being restricted to vascular endothelial cells [32]. Anterior expression of *fli1* was absent in *etsrp* morphants while its tail expression was not affected at the six-somite stage (Figure 5E and 5G). At the ten-somite stage, *fli1* expression in *etsrp* morphants was absent from the anterior and trunk domains (Figure 5F and 5H). As described earlier, angioblasts migrate from the lateral mesoderm towards the midline during somitogenesis [2,3]. *etsrp*-expressing angioblasts were present within the lateral mesoderm but failed to migrate towards the midline in *etsrp* morphants (Figure 5I–5L). Also, no *scl*- or *fli1*-expressing migrating angioblasts were observed in *etsrp* morphants (unpublished data). *etsrp* expression level within the angioblasts was strongly increased in *etsrp* morphants suggesting the presence of negative autoregulation. All of the *etsrp*-expression domains, including the trunk domain, were present in *etsrp* morphants (Figure 5I–5L) (unpublished data).

### Etsrp is Sufficient for the Vascular Endothelial Marker Induction

**Figure 6:**
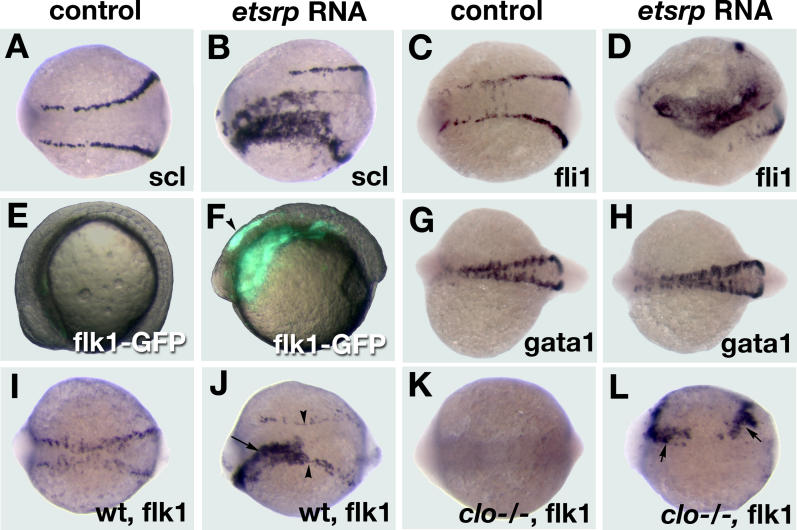
etsrp RNA Overexpression Induces Ectopic Expression of Vascular Endothelial Markers Dorsal view, anterior to the left in all panels except for (E,F) which are lateral views. (A, C, E, and G) Control uninjected embryo; (B, D, F, and H) 100 pg of *etsrp* RNA-injected embryo. (A,B) *scl* expression at the eight-somite stage; (C,D) *flk1* expression at the nine-somite stage. Note the strong ectopic induction of *scl* and *fli1* upon overexpression of *etsrp* RNA. (E,F) Live *flk1*-GFP embryo at the 14-somite stage; fluorescent and transmitted light images were overlayed. Note the very strong ectopic induction of GFP expression in different tissues including neuroectoderm (arrow, F) upon *etsrp* RNA overexpression. Fluorescence in the control uninjected *flk1*-GFP embryo in (E) is not detectable under the same exposure. (G,H) *gata1* expression at the 16-somite stage. Note that *gata1* expression is not affected upon *etsrp* overexpression. (I–L) *etsrp* RNA induces *flk1* expression in *clo* mutant embryos as analyzed using *flk1* probe at the ten- to 12-somite stages. (I) wt (or *clo+/−*) embryo, (J) wt (or *clo+/−*) embryo injected with 100 pg of *etsrp* RNA, (K) *clo−/−* embryo, (L) *clo−/−*embryo injected with 100 pg of *etsrp* RNA. Note that in a *clo+/−* (or wt) embryo *etsrp* RNA induces ectopic *flk1* (arrow, J) in addition to the endogenous *flk1* expression (arrowheads, J) while *clo-/− etsrp* RNA-injected embryo shows only ectopic *flk1* (arrows, L).

We analyzed if *etsrp* mRNA was sufficient for induction of the vascular endothelial markers. Injection of synthetic *etsrp* mRNA into zebrafish embryos at the 1–16 cell stage resulted in strong ectopic induction of vascular endothelial markers *flk1, scl, fli1,* and *cdh5* during somitogenesis (Figure 6A–6F) (unpublished data). Large patches of intense ectopic *flk1* expression were observed within different germ layers including dorsal, lateral, and ventral mesoderm, endoderm and neuroectoderm (Figure 6E and 6F) (unpublished data). No induction of hematopoietic marker *gata1* was observed (Figure 6G and 6H). These results indicate that *etsrp* is sufficient to induce vascular endothelial gene expression in a variety of cell types. Furthermore, *etsrp* specifically induces vascular markers without affecting closely related hematopoiesis.

We tested if *etsrp* was sufficient for vascular induction in *clo−/−* mutant embryos. *etsrp* mRNA was injected into the progeny from *clo+/−* carriers, and the embryos were analyzed for *flk1* expression at the ten- to 12-somite stages. As expected, 25% (23 out of 92) of the uninjected progeny from *clo+/−* carriers showed no *flk1* expression. Among *etsrp* mRNA injected embryos (*n* = 110), 29% displayed normal *flk1* expression pattern (with minor distortions in some embryos), 45% showed both endogenous and ectopic *flk1* expression, 19% showed only ectopic *flk1* expression, and 6% showed no detectable *flk1* expression (Figure 6I–6L). The last two groups apparently represent *clo−/−* homozygous mutants. These results show that the induction of *flk1* by *etsrp* is independent of *clo* function, suggesting that *etsrp* functions downstream of *clo. etsrp* mRNA did not fully restore the endogenous pattern of *flk1* expression in *clo−/−* mutants most likely because it was expressed ubiquitously and not localized to vascular progenitors.

### Etsrp Function Is Required for *flk1* Induction by Vegf and Scl

**Figure 7:**
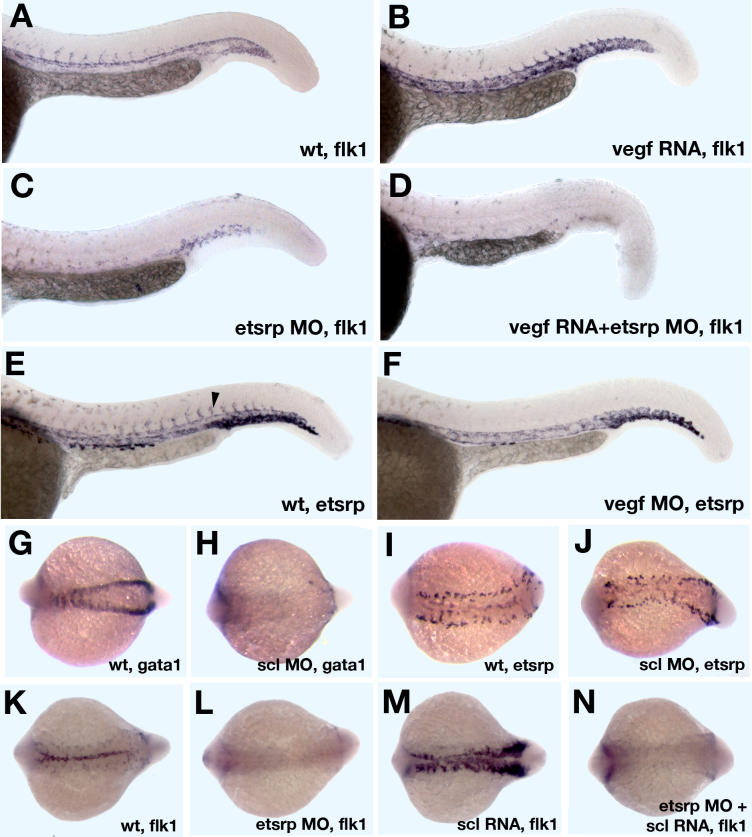
etsrp Is Required for *vegf* and *scl* Signaling (A–D) Etsrp is required for Vegf signaling as assayed for *flk1* expression at 26 hpf. (A) Control uninjected embryo, (B) *vegf* RNA-injected embryo, (C) 7.5 ng of *etsrp* MO2-injected embryo, (D) *vegf* RNA- and *etsrp* MO2-co-injected embryo. Note that *vegf* RNA induces strong *flk1* expression in (B) while *vegf* RNA and *etsrp* MO co-injection results in loss of *flk1* expression in (D), similar to the *etsrp* morphant phenotype in (C). (E,F) Etsrp expression analysis in Vegf morphants at 26 hpf. (E) Control uninjected embryo; (F) 10.5 ng of *vegf* MO-injected embryo. Note that *vegf* morphants have lost *etsrp* expression in the intersegmental vessels (arrowhead, E). (G–J) Scl knockdown affects *gata1* but not *etsrp* expression at the 15-somite stage. Dorsal view, anterior is to the left. (G,I) Control uninjected embryo; (H,J) 10 ng *scl* UTR-MO-injected embryo. (G,H) *gata1* expression; (I,J) *etsrp* expression. (K–N) Etsrp is required for *scl* signaling in *clo* mutants as analyzed for *flk1* expression at the 15-somite stage. (K) Control uninjected embryo; (L) 7.5 ng *etsrp* MO2-injected embryo; (M) *scl* RNA-injected embryo; (N) *scl* RNA- and *etsrp* MO2-co-injected embryo. Note that *scl* RNA causes ectopic *flk1* expression in (M) which is lost upon knockdown of Etsrp in (N).

We analyzed the epistatic relationship between *vegf* and *etsrp. vegf* overexpression has been reported to induce strong expression of vascular markers such as *flk1* [43] (Figure 7A and 7B). Co-injection of *vegf* mRNA together with *etsrp* MO resulted in the loss of *flk1* expression, similar to *etsrp* MO phenotype, indicating that *etsrp* function is required for *flk1* induction by *vegf* signaling (Figure 7C and 7D). Downregulation of *vegf* expression resulted in the loss of *etsrp* expression in the intersegmental vessels (Figure 7E and 7F). *etsrp* expression in dorsal aorta was also not apparent while cardinal vein seemed expanded. This is consistent with the previous report of *vegf* involvement in regulating arterial fate in zebrafish [44]. Expression of *etsrp* was not affected in *vegf* morphants during mid-somitogenesis stages (unpublished data).

Scl has been implicated in the hematopoietic and vascular development in zebrafish [13,14]. Downregulation of Scl function using two different MOs had no significant effect on the *etsrp* expression in angioblasts while the hematopoietic expression of *gata1* was completely eliminated (Figure 7G–7J). Overexpression of *scl* mRNA has been reported to cause induction of vascular markers [45]. We tested if *etsrp* MO would block this induction by co-injecting it together with *scl* mRNA. The embryos were analyzed for *flk1* expression at the 15-somite stage (Figure 7K–7N). As expected, no or only extremely weak *flk1* expression was observed in *etsrp–*MO-injected embryos (Figure 7L). Overexpression of *scl* mRNA caused strong induction of *flk1* (Figure 7M). Embryos, co-injected with *scl* mRNA and *etsrp* MO showed no or very weak *flk1* expression (Figure 7N). These results demonstrate that *etsrp* function is required for *flk1* induction by *scl.*

## Discussion

In this study, we report characterization and functional analysis of a novel zebrafish Ets1-related protein, Etsrp. Knockdown of Etsrp resulted in the complete absence of functional blood vessels and downregulation of all endothelial-specific markers analyzed. In zebrafish, *hedgehog* signaling is necessary for migration of angioblasts from the lateral mesoderm toward the midline, where they subsequently differentiate within the ICM region [4]. Such migration is not apparent in the anterior region which gives rise to the anterior vessels including head vessels. Interestingly, vasculogenesis defects in *etsrp* morphants were more pronounced in the posterior region of the embryo which was particularly evident at lower downregulation levels. At high MO doses, endothelial cells were nearly completely absent from axial and intersegmental vessels. However, head expression of endothelial markers was not completely eliminated. It is possible that the remaining amount of Etsrp protein activity is sufficient for the head vasculogenesis. Upregulation of *etsrp* RNA was particularly strong in the head region in *etsrp* morphants, which may partially compensate for the MO-mediated translation inhibition. Alternatively, *etsrp* function may be redundant in the vasculogenesis of head vessels.

Among the vascular-specific genes analyzed, expression of only *fli1* and *scl* was not globally downregulated in the early embryos. The early *fli1* expression, which overlaps with the hematopoietic factor *gata2* expression, is unaffected in the *clo* mutants and *scl* morphants and has been suggested to mark the common blood and vascular precursors, hemangioblasts [13,32]. *fli1* is later expressed specifically in the vascular endothelial cell precursors, and this expression was reduced but not completely eliminated in *etsrp* morphants. *fli1* expression may not be directly regulated by *etsrp,* therefore undifferentiated angioblasts retain some of *fli1* expression in *etsrp* morphants. *scl* is expressed in both hematopoietic and vascular progenitors throughout most of the early development. Although we did not see global downregulation of *scl* expression in *etsrp* morphants, it is likely that *scl* expression is lost in angioblasts but retained in hematopoietic precursors. Interestingly, expression of both *fli1* and *scl* genes was lost from the anterior and trunk domains of the lateral mesoderm. Anterior domain contains angioblast and myeloid progenitors, which appear to be absent from the *etsrp* morphants. The difference between the trunk and posterior lateral mesoderm has not been previously analyzed in great detail. Possibly, the trunk region contains mostly angioblasts, while the tail region contains both angioblasts and hematopoietic precursors.

Overexpression of *etsrp* induced strong ectopic expression of vascular markers in multiple cell types, including even tissues that normally commit to a very different fate such as neuroectoderm. Furthermore, overexpression of *etsrp* specifically initiated vascular development without affecting related hematopoiesis, which argues that the observed effects are not caused by an early ventralization of the whole embryo. These results indicate that *etsrp* is sufficient to initiate vasculogenesis. As a contrast, overexpression of other regulators of vasculogenesis such as *vegf* and *scl* induced expression of vascular markers only within the lateral or somitic mesoderm [14,43,45].

Overexpression of *vegf* and *scl* did not have an effect on vascular development in the absence of *etsrp* function, which suggests that *etsrp* is an essential mediator of *vegf* and *scl* signaling, at least in the vascular induction. Loss of *vegf* or *scl* did not affect early expression of *etsrp* within angioblasts. Overexpression of *etsrp* caused strong *scl* induction, indicating that *etsrp* plays an important role in controlling *scl* expression, at least within angioblasts. This is also supported by the loss of *scl* expression in the anterior and trunk regions in *etsrp* morphants. Our data suggest that *etsrp* is necessary for *scl* expression within angioblasts, and both genes are then required for induction of multiple vascular endothelial genes.

The current study shows for the first time that a single gene can be necessary and sufficient for initiating vascular development in vertebrates. Our results demonstrate that Etsrp acts as a very early regulator of vasculogenesis. These findings will greatly advance our understanding of vascular development and the general mechanisms of cell fate specification.

## Materials and Methods

### Microinjection of MOs

Two *etsrp*-specific MOs (MO1, TTGGTACATTTCCATATCTTAAAGT and MO2, CACTGAGTCCTTATTTCACTATATC; Gene Tools, Inc.) were used to inhibit the function of Etsrp protein. For the dose response curve, 1–6 nl of 1 mg/ml MO solution in the Danieau buffer supplemented with 15 mM Tris-Cl (pH 7.5) was injected into one- to two-cell-stage embryos [36]. For phenotypic and marker analysis, 3–4nl of 2.5 mg/ml MO2 solution was injected. A five-base MO2 mismatch ( CAGTGAGACCTTAATTCAGTATAAC) was used as a control MO. A previously described Vegf-A-1 MO (kindly donated by S.C. Ekker) was used to inhibit Vegf function [46]. Scl translation-blocking UTR-MO ( GCTCGGATTTCAGTTTTTCCATCAT) and previously described splice-blocking MO [13] were used to inhibit the function of Scl protein. Microinjections were performed as described [47].

### BAC modification and microinjection

CHORI-211 BAC library from Children's Hospital Oakland Research Institute, Oakland, California, United States was used to identify *etsrp*-containing–BAC of approximately 250 kilobases. Replacement of Etsrp coding sequence with GFP was essentially performed as described [38]. Purified BAC DNA (100–150 pg) was injected into blastomere at the one-cell stage.

### RNA overexpression and epistasis experiments

Etsrp overexpression construct was generated by subcloning the open reading frame of etsrp into the SpeI site of pT3TS [47]. To synthesize mRNA, Etsrp-T3TS was linearized with XbaI and transcribed using T3 mMessage mMachine Kit (Ambion, Austin, Texas, United States). At the two- to 16-cell stage for overexpression studies, 75–15 pg of etsrp mRNA was injected into zebrafish embryos. For epistasis studies, approximately 50 pg of VEGF121 and VEGF165 mRNA 1:1 mixture was injected [43]. Approximately 300 pg of scl mRNA was injected for epistasis studies [12].

### In situ hybridization

In situ hybridization was performed as described [48]. The following probes were used: *etsrp* [42], *flk1* [40], *fli1* [40], *flt4* [40], *scl* [12], *gata1* [49], *admr* [42], *cdh5* [42], *crl* [42].

### Analysis of *etsrp* knockdown phenotype

o-dianisidine heme staining was performed as described [49]. Microangiography was performed as described [46].

### Zebrafish strains

Majority of *etsrp* knockdown analysis was performed in the wild-type zebrafish from Scientific Hatcheries (Huntington Beach, California, United States). As confirmed by sequencing, no polymorphisms in the *etsrp* MO-binding sites were detected in this wild-type strain. In addition, *flk1*-GFP transgenic zebrafish line was used [39]. *clo^m39^* line was used in *etsrp* overexpression experiments [7].

## Supporting Information

Figure S1 Etsrp MO Blocks GFP Expression in the Embryos Injected with *etsrp*–GFP-Modified BAC Construct Mosaic panels have been generated by taking individual images of randomly chosen embryos from each batch. Embryos are at the 90% epiboly stage. (A) Following microinjection, *etsrp*-GFP BAC is transiently expressed in a mosaic pattern. Note that the transient BAC expression commonly does not recapitulate the endogenous expression pattern. (B) Co-injection of *etsrp*-GFP BAC and 10 ng of *etsrp* MO2 completely eliminated GFP fluorescence. (C) Co-injection of *etsrp*-GFP BAC and 10 ng of 5-base MO2 mismatch had no effect on GFP fluorescence.
(2.5 MB TIF).

### Accession Numbers

GenBank (http://www.ncbi.nlm.nih.gov/Genbank) accession number for *etsrp* is DQ021472.

## Abbreviations

BAC: bacterial artificial chromosome
dpf: days-post-fertilization
GFP: green fluorescent protein
hpf: hours-post-fertilization
ICM: intermediate cell mass
MO: morpholino
Vegf: vascular endothelial growth factor
wt: wild-type
